# Specialized multi-disciplinary heart failure clinics in Ontario, Canada: an environmental scan

**DOI:** 10.1186/1472-6963-12-236

**Published:** 2012-08-03

**Authors:** Harindra C Wijeysundera, Gina Trubiani, Lusine Abrahamyan, Nicholas Mitsakakis, William Witteman, Mike Paulden, Gabrielle van der Velde, Kori Kingsbury, Murray Krahn

**Affiliations:** 1 Division of Cardiology, Schulich Heart Centre, Sunnybrook Health Sciences Centre, Ontario, Canada; 2 Toronto Health Economics and Technology Assessment (THETA) Collaborative, Ontario, Canada; 3 Department of Medicine, University of Toronto, Ontario, Canada; 4 Institute for Work & Health, Ontario, Canada; 5 Cardiac Care Network of Ontario, Ontario, Canada; 6 University Health Network – Toronto General Hospital, Ontario, Canada; 7 Faculty of Pharmacy, University of Toronto, Ontario, Canada

## Abstract

**Background:**

Multi-disciplinary heart failure (HF) clinics have been shown to improve outcomes for HF patients in randomized clinical trials. However, it is unclear how widely available specialized HF clinics are in Ontario. Also, the service models of current clinics have not been described. It is therefore uncertain whether the efficacy of HF clinics in trials is generalizable to the HF clinics currently operating in the province.

**Methods:**

As part of a comprehensive evaluation of HF clinics in Ontario, we performed an environmental scan to identify all HF clinics operating in 2010. A semi-structured interview was conducted to understand the scope of practice. The intensity and complexity of care offered were quantified through the use of a validated instrument, and clinics were categorized as high, medium or low intensity clinics.

**Results:**

We identified 34 clinics with 143 HF physicians. We found substantial regional disparity in access to care across the province. The majority of HF physicians were cardiologists (81%), with 81% of the clinics physically based in hospitals, of which 26% were academic centers. There was a substantial range in the complexity of services offered, most notably in the intensity of education and medication management services offered. All the clinics focused on ambulatory care, with only one having an in-patient focus. None of the HF clinics had a home-based component to care.

**Conclusions:**

Multiple HF clinics are currently operating in Ontario with a wide spectrum of care models. Further work is necessary to understand which components lead to improved patient outcomes.

## Background

Heart failure (HF) is a complex, progressive syndrome characterized by abnormal heart function resulting in poor exercise tolerance, recurrent hospitalizations, and reductions in both quality of life, and survival [1]. Although tremendous progress has been made in pharmacologic and device therapy, HF patients continue to have a poor prognosis, with an annual mortality ranging from 5% to 50% [1]. The incidence of HF is projected to increase, with estimates suggesting a three-fold increase in HF hospitalizations over the next decade [2]. Alternative targeted health care delivery models have therefore been of particular interest in HF, as a means of improving both quality of life and survival [3].

Disease management through multi-disciplinary community care clinics has been shown to improve patient outcomes in different health conditions, including diabetes, chronic kidney disease, and cancer [4,5]. The potential benefits of a multi-disciplinary strategy in HF include improved utilization and adherence with evidence-based medications. This model of care may also address the complex interplay between medical, psychosocial, and behavioural factors facing these patients and their caregivers [3]. Multiple previous randomized studies and meta-analyses have evaluated the efficacy of such clinics with some suggesting a reduction in mortality in excess of 20% [1,3,6]. However, interpreting this literature is challenging because of substantial heterogeneity in the composition of the HF clinics, the interventions they offer, and the population studied [3,7].

Currently, specialized HF clinics do not receive specific funding from the Ontario Ministry of Health and Long Term Care (MOHLTC), the third party payer for government insured health services in the province. It is not known how widely available specialized HF clinics are in Ontario, nor has their composition, or the services they offer, been described. Therefore, it is unclear if the efficacy of HF clinics in randomized trials is generalizable to the HF clinics currently in place in Ontario. Our objective was to address these important gaps in knowledge, through a comprehensive field evaluation, whereby real world practice for HF patients in Ontario was assessed in 2010. Specially, we aimed to understand the current availability of specialized HF clinics in the province, and the intensity and complexity of services offered.

## Methods

Canada is divided into 13 distinct territories or provinces, with Ontario being the most populous. Based on the most recent census, 12.2 million of Canada’s 31.6 million people lived in Ontario. The Ontario population is concentrated around major urban areas, with only 15% living in rural settings, defined as a population less than 1000 persons and less than 400 persons per km^2^.

There is universal access to medical care in Canada without user-fees or out-of-pocket payments. Health care funding is determined at the provincial level. In 2006, the Ontario Ministry of Health and Long-Term Care transferred the responsibility for planning, integrating and funding of health services within the province to 14 regional Local Health Integration Networks (LHIN).

### Identification of Heart Failure Clinics

For the purpose of this project, a specialized HF clinic was defined as a clinic that consists *at a minimum* of a physician and a nurse, one of whom has specialized training/interest in HF. This definition is consistent with that used in recent systematic reviews of HF clinics [8].

We utilized three approaches to identify clinics. First, all hospitals listed on the MOHLTC site (http://www.health.gov.on.ca) were contacted. Notices were posted in the Cardiac Care Network (CCN) webpage. Finally, we used snow-ball sampling, an approach often used in qualitative or mixed methods research studies, to evaluate ‘hidden populations’ [9].

A hidden population is one in which a sample frame (i.e. a list of all the members of the population) cannot be constructed, thereby preventing probability sampling [9]. An alternative that does not require a sampling frame is snow-ball sampling, whereby new members are selected from the social network of existing members of the sample [9].

In this method, a number of seeds are first selected [9]. These seeds are members of the hidden population that have been identified. The seeds are interviewed and form stage 0 of the sampling process. The seeds identify other members of the population, who are in turn approached in the next generation of sampling (stage 1). This process is continued until the desired sample size is reached. This method has been successfully utilized in a myriad of cardiac studies [10-12].

In our study, the initial seeds were the Ontario members of the Canadian Heart Failure Network (CHFN) and other sites identified by the expert panel (Table 1). Established in 1999, the CHFN is a network of academic and community based clinics that provide specialized care to HF patients (http://www.cfna.ca). Importantly, the network did not include all HF clinics in the province, thereby necessitating further sampling.

**Table 1:** Seed heart failure clinics

**Clinic name and location**	
1.	Cornwall: Cornwall Community Hospital
2.	Hamilton: Heart Function Clinic - Hamilton Health Sciences Corporation
3.	Kingston: Hotel Dieu Hospital
4.	Kitchener: St. Mary's Hospital
5.	London: London Health Sciences Centre
6.	Oakville: Oakville-Trafalgar Memorial Hospital
7.	Orillia: Orillia Soldiers' Memorial Hospital
8.	Ottawa: University of Ottawa Heart Institute
9.	Owen Sound: Grey Bruce Health Services
10.	Picton: Prince Edward Family Health Team Heart Failure Clinic
11.	Toronto: University Health Network (UHN) (1)
12.	Toronto: University Health Network (UHN) (2)
13.	Toronto: Mt Sinai Heart Function Clinic
14.	Toronto: St Michael’s Hospital Heart Function Clinic
15.	Toronto: Sunnybrook Hospital Heart Function Clinic

The physician or nursing lead at each clinic was approached and a semi-structured interview conducted to establish the scope of the practice. The lead was asked to identify any other HF clinics, which may serve patients in the vicinity (1^st^ sampling stage). We continued to accrue new sampling stages until no new clinics were identified, at which point the sample was saturated.

### Regional differences in access to HF Clinics

The boundaries of each LHIN were used to assess any geographic inequalities in access to HF clinics. We first determined the population size overall and of persons greater than the age of 65 years in each LHIN. The number of prevalent HF cases in each LHIN is not known. To approximate the burden of HF per LHIN, we used previously published data on the number of hospital discharges per LHIN with a most responsible diagnosis of HF in the fiscal years 1997–2001 [13]. We then determined the annual rate of HF hospital discharges per HF clinic in each LHIN as another estimate of the regional distribution of access to care.

### Semi-structured Interview

The semi-structured interview ascertained information broadly on the characteristics of the clinics themselves and the program service model. We used the HF Disease Management Scoring Instrument (HF-DMSI), a validated questionnaire developed by Riegel and colleagues to measure the intensity and complexity of each clinic’s program service model across 10 categories [14]. Details on the categories and the respective scoring algorithm are found in Table 2[14]. Two researchers (GT, WW) independently scored each clinic based on the interview transcripts.

**Table 2:** Heart failure disease management scoring instrument (HF-DMSI)

** Intervention category**	** Points to be assigned**
**Recipient**	1 = Provider alone
	2 = Patient alone
	3 = Patient with some inclusion of caregiver
	4 = Patient with a caregiver who is central to the intervention
**Intervention content**	
**Education and counselling aimed at supporting self-care**	0 = No mention of education
	1 = Focus solely on importance of treatment adherence
	2 = Focus on treatment adherence including some creative methods of improving adherence
	3 = Focus on surveillance but no mention of actions to be taken in response to symptoms (eg, no flexible diuretic management)
	4 = Emphasis on surveillance, management, and evaluation of symptoms in addition to treatment adherence
**Medication management**	0 = No mention of medication regimen
	1 = Some mention of medications (eg, importance of medication compliance) but not an active part of the intervention. No attempt to intervene with provider to get patients on an evidence-based medication regimen
	2 = Evidence-based medication regimen advocated but no follow-up with patient or provider to monitor the suggestion
	3 = Medication regimen monitored, attempt made to get the patient on evidence-based medications, with follow-up monitoring done with patient or provider
**Social support Peer support**	0 = No mention of a peer support intervention
	1 = Peer support mentioned but not integral to intervention
	2 = Peer support integral component of intervention
**Surveillance by provider:** **Remote monitoring**	0 = No use of remote monitoring or telehealth
	1 = Remote monitoring is used in conjunction with other interventions that form the main intervention used
	2 = Telehealth is essential component of intervention
**Delivery personnel**	1 = Single generalist provider (eg, physician, nurse, pharmacist)
	2 = Single HF expert provider (eg, physician, nurse, pharmacist)
	3 = Multidisciplinary intervention
**Method of communication**	1 = Mechanized via internet or telephone
	2 = Person-to-person by telephone
	3 = Face-to-face, individual, or in a group
	4 = Combined: Face-to-face at least once alone or in a group with individual telephone calls in between meetings
**Intensity and complexity**	
**Duration**	1 = ≤1 mo
	2 = ≤3 mo
	3 = ≤6 mo
	4= > 6 mo
**Complexity**	1 = Low: single contact with little or no follow-up
	2 = Moderate: >1 but <4 and/or infrequent contact or contacts of short duration
	3 = High: multiple contacts of significant duration
**Environment**	1 = Hospital: Inpatient only
	2 = Clinic/outpatient setting
	3 = Home-based
	4 = Combination of settings

Briefly, the HF-DMSI focused on the composition of the HF team (single practitioner vs. multi-disciplinary team) and the content of the HF intervention such as education (scored from 0 to 4, with 4 as the more comprehensive education program), and medication management (scored from 0 to 3). The environment of the HF clinics was categorized as those that only focused on inpatients with HF (score of 1) versus those that focused only on outpatients seen in clinic (score of 2), those that were home-based with the intervention taking place in the patients’ residence (score of 3), with clinics that had components in more than one setting receiving the highest score of 4. Peer support, remote monitoring, and the duration and complexity of contact were also measured. The instrument was designed to provide a separate score for each category. The HF-DMSI has content validity and an excellent inter-rater reliability with a intra-class correlation coefficient of 0.918 [14].

Because the HF-DMSI does not provide an overall summary score, and could not be used to rank clinics, we performed a concept mapping exercise, using an HF expert panel. The concept mapping exercise consisted of two parts [15,16]. In part 1, we determined the relative importance of each of the 10 categories of the HF-DMSI, based on consensus of the expert panel. In the second part, each of the clinics identified were categorized by the expert panel into three intensity groups, based on their scores on the HF-DMSI, influenced by the implicit weighting system revealed in part 1. Further description of this process is found in Additional file 1 Appendix A.

### Institutional review board

The ethics review board of the University of Toronto approved this protocol. When required by local institutional regulations, separate institutional review board approval was acquired for each participating clinic. Consent for the use of the structure survey results was obtained from the physician lead for each identified HF clinic.

## Results

### HF clinic identification

**Figure 1:**
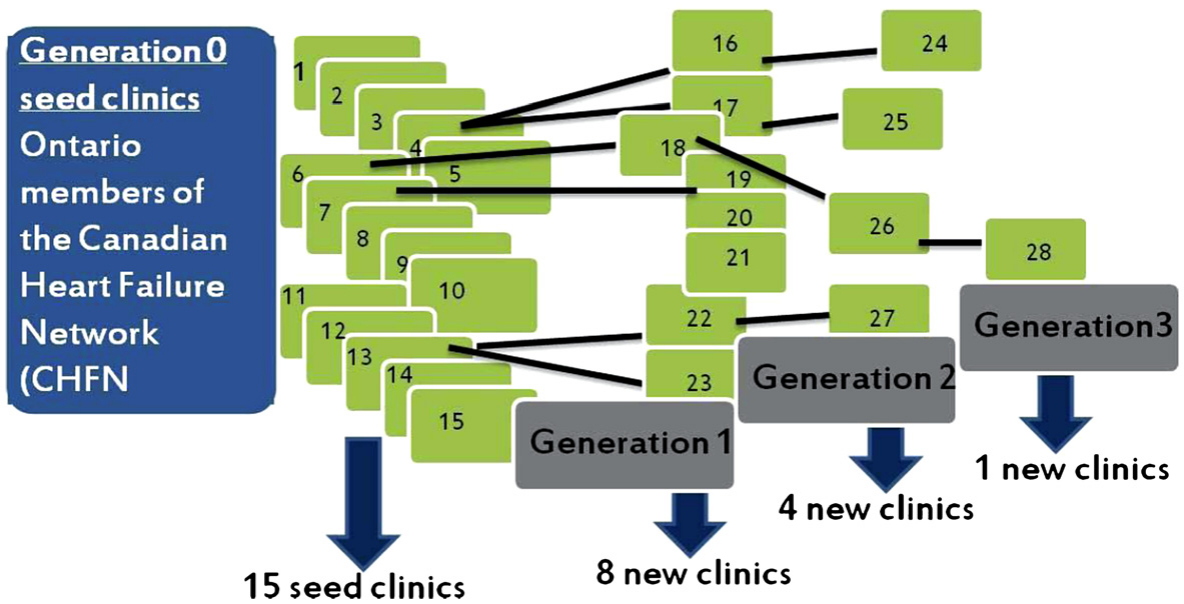
**Process by which 28 clinics were identified by snowball sampling.** Based on interview responses from the initial 15 seed clinics, full saturation was reached in 3 generations.

Between May 2010, and August 2010, we identified a total of 34 clinics through our sampling method, as seen in Figure 1. From the initial 15 seed clinics identified through the CHFN, three generations of snow-ball sampling took place, at which point the sample was saturated. Five clinics were identified through the CCN and one HF clinic through contacting individual hospitals. Of these clinics, 30 agreed to participate in the semi-structured survey.

### Regional distribution of HF clinics

**Figure 2:**
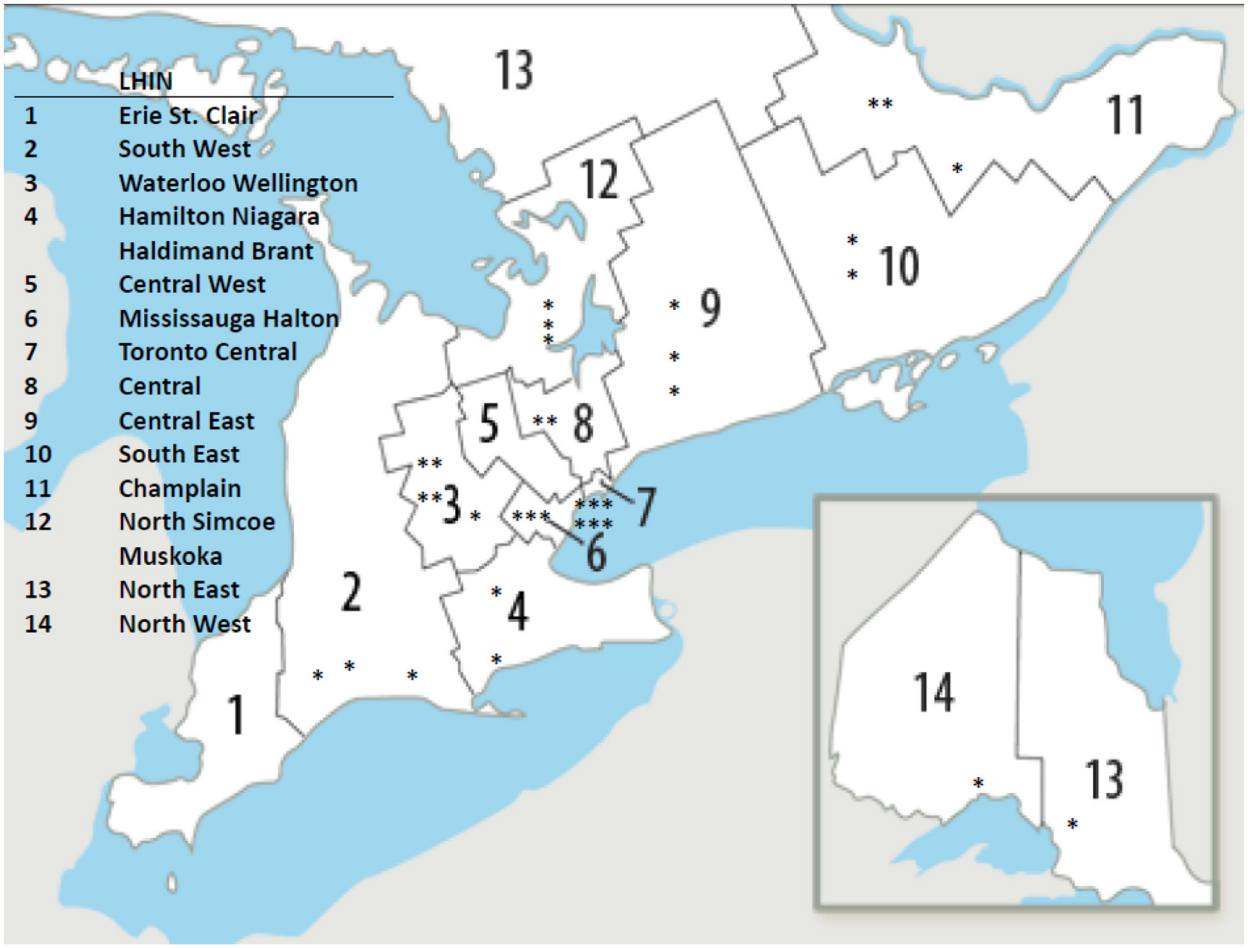
Regional Local Health Integration Networks (LHIN) in Ontario depicting regional distribution of identified heart failure clinics.

The initial seed clinics were located in 9 of the Ontario 14 LHIN’s. We were able to identify HF clinics in all the remaining LHINs except for the Central West and Erie St Clair LHINs. There was substantial regional variation in access to HF clinics. As apparent from Figure 2 and Table 3, the identified HF clinics were concentrated in the south and central regions of the province. Each HF clinic served an average population of 353,800 with an over 65-year-old population of 45,200. However, there was a substantial range in the population served in the LHINs with identified HF clinics, from 179,200 per clinic in the Toronto Central LHIN, to 761,400 in the central LHIN.

**Table 3:** Geographic distribution of clinics

** LHIN**	**# HF Clinics**	**Total Population**	**population per HF Clinic**	**age 65 years and over in LHIN**	**>65 years population per HF clinic**	**annual HF discharge per HF clinic**
Erie St. Clair	0	623,300	NA	85,000	NA	NA
South West	3	890,100	296,700	125,800	41,900	247
HNHB	2	1,298,300	649,100	192,400	96,200	591
Waterloo Wellington	5	679,700	135,900	76,000	15,200	84
Mississauga Halton	3	1,002,300	334,100	103,400	34,500	155
Central West	0	735,200	NA	65,900	NA	NA
Central	2	1,522,800	761,400	183,100	91,600	395
Central East	3	1,419,800	473,300	184,600	61,500	305
Toronto Central	6	1,075,100	179,200	131,800	22,000	118
North Simcoe Muskoka	3	417,000	139,000	59,900	20,000	108
South East	2	457,200	228,600	74,700	37,400	217
Champlain	3	1,131,400	377,100	137,600	45,900	247
North East	1	545,000	545,000	84,900	84,900	626
North West	1	231,900	231,900	31,400	31,400	218
Total	34	12,028,900	353,800	1,536,500	45,200	200

*LHIN*: Local Health Integration Network; *HF*: Heart Failure; *HNHB*: Hamilton Niagara Haldimand Brant; *NA*: not applicable.

In order to estimate the burden of HF across Ontario, we used data which showed over the 5 years from 1998 to 2002, 42,367 patients were discharged with a diagnosis of HF. As seen in Table 3, given the 34 clinics, on average each HF clinic would be able to serve 200 HF discharges per year. However, there was substantial regional variability, with greater than a 7fold difference between LHINs with HF clinics. For example in Waterloo, there was a HF clinic for 84 HF discharges, compared to one HF clinic per 626 HF discharges in the North East LHIN.

### Clinic characteristics

**Figure 3:**
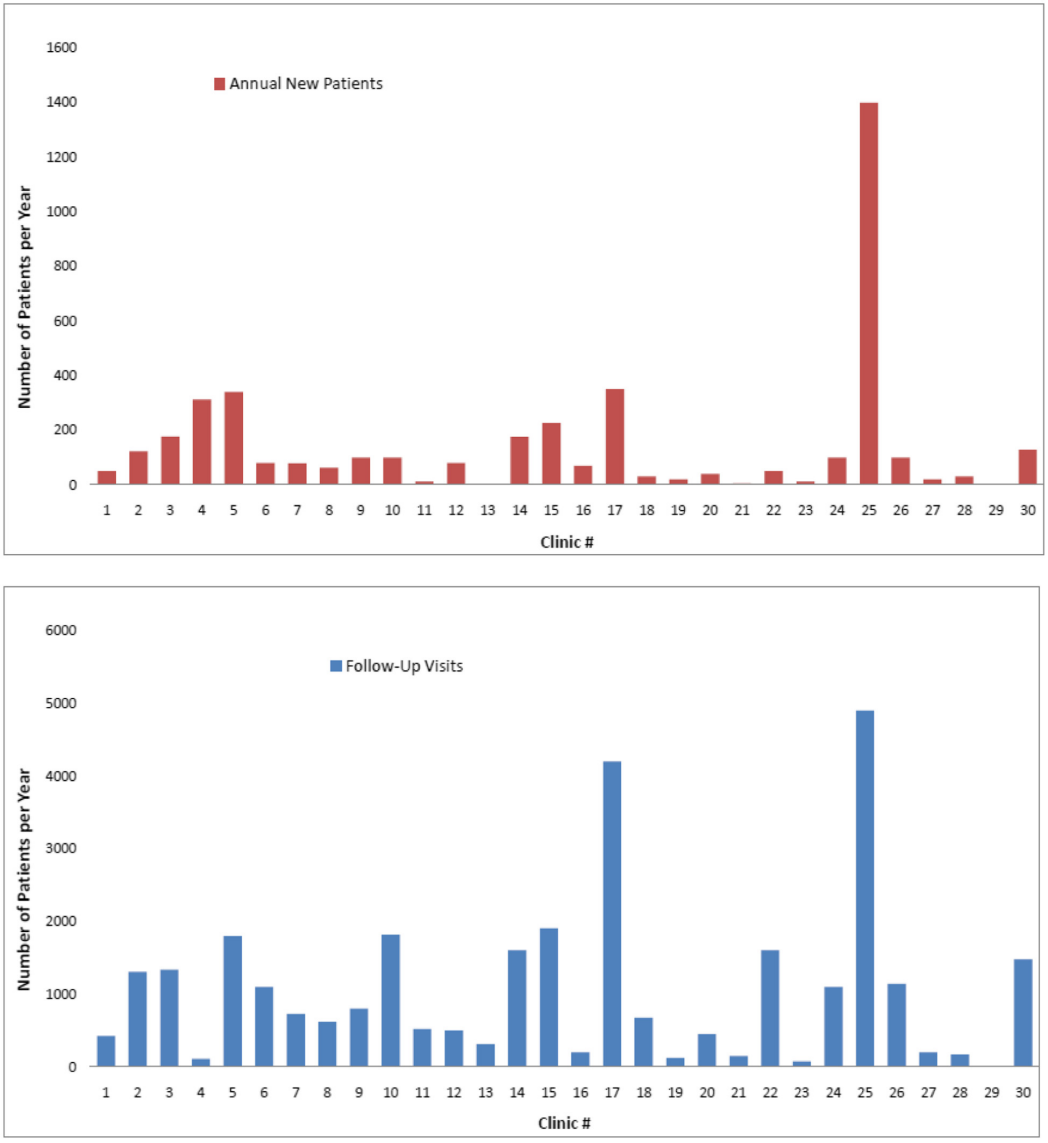
**Annual Service Volume of the identified Heart Failure Clinics.** The red bar indicates new patients per year, and the blue bar represents annual patient visits.

Identified HF clinics had a mean of 138 new consults (median 78; interquartile range 25–128) and 1020 visits per year (median 675; interquartile range 200–1479). However, there was substantial variation in their service volume, as evidence in Figure 3, with two high volume clinics which were outliers (clinic #17 and #25). Clinic #25 had 4900 annual visits, with 1400 new patients per year. Clinic #17 had 4200 annual visits, but only 350 new patients annually. In contrast to the other HF clinics, the majority of patients seen at clinic 4 were new (represented by the red bar), with only a limited number of follow-up visits (represented by the blue bar).

The majority (80.6%) of clinics were physically based in hospitals with 25.8% being part of an academic institution. In total, 143 HF clinic physicians worked at the 30 identified clinics. The majority of clinics were run by cardiologists.

### Access to allied health professionals

The clinics had on average limited access to in-clinic allied health professionals, as seen in Table 4. Under half had access to dieticians or pharmacists, with only 6.5% and 16.1% with in-clinic access to physiotherapists or counsellors. 87.1% of HF clinics had a formal affiliation with a cardiac rehabilitation program and 64.5% where actively involved with chronic disease management of another condition, such as diabetes mellitus.

**Table 4:** Characteristics of 30 identified clinics

** Parameter**	
**PERSONEL**	
Mean number of Physicians	4.7 (1–8)*
% of clinics with cardiologist	80.6
% of clinics with internists	22.6
% of clinics with family physicians	9.7
% of physicians with heart failure training	80.6
Mean Number of Nurses	2.0 (1–6)*
**LOCATION**	
% Academic	25.8
% Community Based	74.2
Mean Annual Total Visits	1020 (200–1479)*
Mean Annual Total New Patients	139 (25–128)*
% Access to Onsite Echocardiography	80.6
% Access to Onsite Nuclear Cardiology Testing	58.1
% Access to Onsite Angiography	38.7
% Access to Onsite exercise Stress Testing	77.4
Mean Exam Rooms	3.3 (1–4)*
**ALLIED HEALTH PROFESSIONALS**	
% Access to Dietician (In Clinic)	45.2
% Access to Pharmacist (In Clinic)	32.3
% Access to Physiotherapy (In Clinic)	6.5
% Access to Counselor (In Clinic)	16.1
% Affiliated with Cardiac Rehabilitation	87.1
% Involved in other Chronic Disease Management	64.5

* inter-quartile range is shown.

### Intensity and complexity

**Figure 4:**
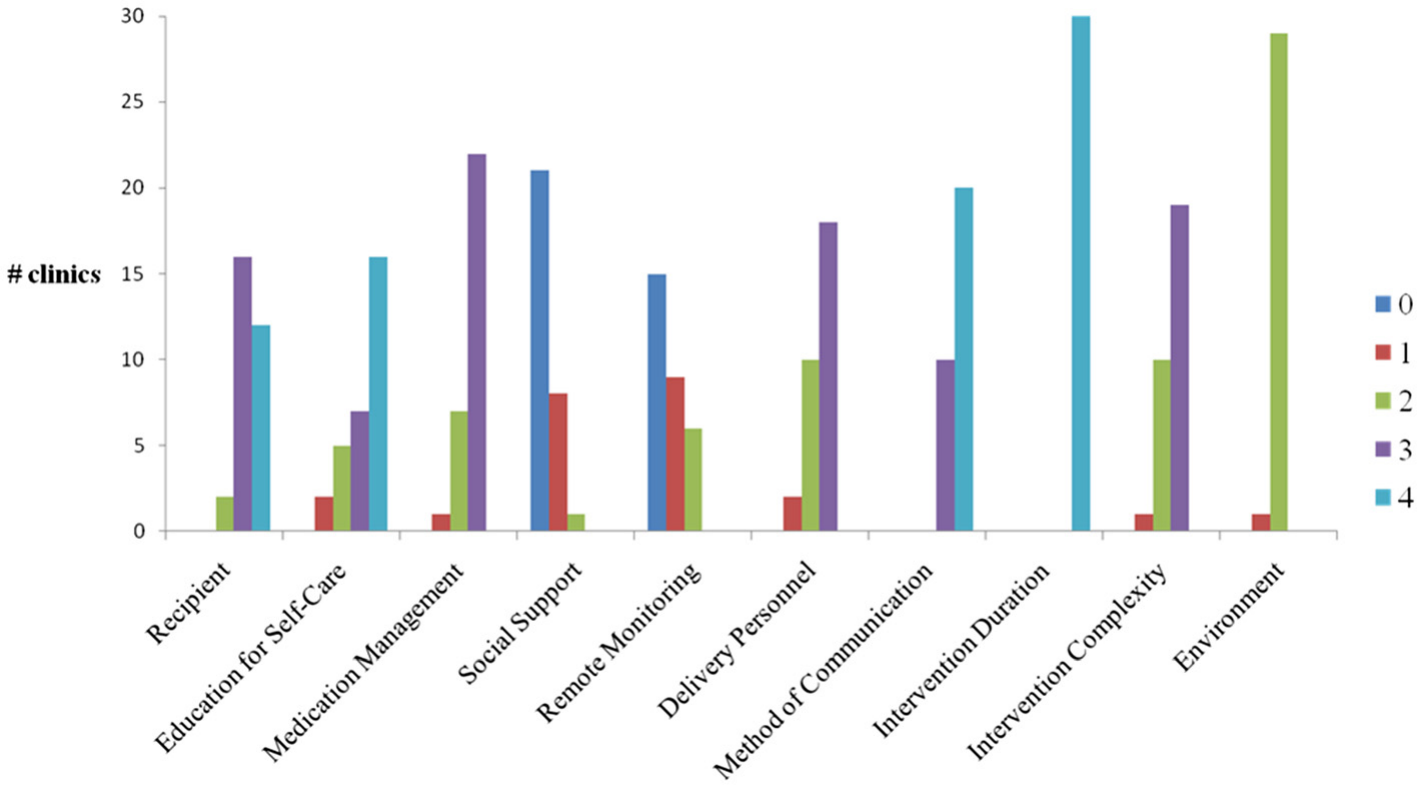
**Distribution of scores on 10 categories of Heart Failure Disease Management Scoring Instrument (HF-DMSI).** Please refer to Table 2 for specific definitions of individual scores. Higher scores indicate more comprehensive program within that category.

The ranges of HF clinic scores on the HF-DMSI are shown on Figure 4. There was little variation between the clinics for some elements of the instrument, such as intervention duration (all scored 4; greater than 6 months). The majority of HF clinics had a formal medication management protocol, where medications were monitored and an attempt was made to increase utilization of evidence-based medications. There was substantial range in the intensity of education and counselling aimed at supporting self-care. Although all clinics had some form of education program, these ranged from programs that focused only on adherence to more comprehensive programs that emphasized surveillance, management and evaluation of symptoms in addition to treatment adherence. The majority of clinics did not use remote monitoring at the clinic, although half did contact patients by telephone in between face-to-face evaluations. A formal peer support component was identified in only one HF clinic. Somewhat surprisingly, although the delivery personnel at the clinic were multidisciplinary in approximately 50% of clinics, some had only either a single generalist or HF expert provider. As far as environment, all of the clinics were ambulatory based, with one that was predominantly focussed on inpatients. None were exclusively home-based or had a home-based component.

### Concept mapping

Based on our concept mapping exercise, the expert panel categorized the 30 identified clinics into three strata of intensity; 8 clinics were assigned to the low intensity category, with 12 in the medium intensity category and 10 in the high intensity group. The mean scores on the HF-DMSI for these three strata are shown in Table 5. Although the high intensity clinics had higher mean scores in 9 of the 10 HF-DMSI categories, this was most pronounced in the education and counselling, medication management, delivery personnel and complexity categories. This suggests an implicit weighting of these categories by our expert panel as revealed by the concept mapping exercise. In contrast, remote monitoring and the presence of a structured peer-support program were believed to be of lesser importance.

**Table 5:** Clinic intensity and complexity

** HF-DMSI category**	**All clinics** **(n = 30)**	**Clinic intensity types**			
		**High**	**Medium**	**Low**	**p-value**
		**(n = 10)**	**(n = 13)**	**(n = 7)**	
Recipient	3.3 ± 0.6	3.7 ± 0.5	3.2 ± 0.6	3.0 ± 0.6	.040
Education and counselling aimed at supporting self-care	3.2 ± 1.0	3.9 ± 0.3	3.1 ± 1.0	2.6 ± 1.1	.011
Medication management	2.7 ± 0.5	3.0 ± 0	2.8 ± 0.4	2.1 ± 0.7	.002
Peer support	0.3 ± 0.5	0.6 ± 0.7	0.2 ± 0.4	0.3 ± 0.5	.147
Remote monitoring	0.7 ± 0.8	1.0 ± 0.8	0.8 ± 0.8	0.1 ± 0.4	.079
Delivery personnel	2.5 ± 0.6	3.0 ± 0	2.5 ± 0.5	2.0 ± 0.8	.002
Method of communication	3.6 ± 0.5	4.0 ± 0	3.5 ± 0.5	3.4 ± 0.5	.018
Duration	4.0 ± 0	4.0 ± 0	4.0 ± 0	4.0 ± 0	-
Complexity	2.6 ± 0.6	3.0 ± 0	2.6 ± 0.5	2.0 ± 0.6	<.001
Environment	2.0 ± 0.2	2.0 ± 0	1.9 ± 0.3	2.0 ± 0	.536

*HF-DMSI* : Heart Failure Disease Management Scoring Instrument. Results are presented as means ± standard deviations. Please refer to Table 2 for detail description of HF-DMSI categories and scoring.

## Discussion

In this environmental scan of HF clinics in the province of Ontario, Canada, we were successfully able to identify 34 HF clinics. There was substantial inequity in access to care, with two LHINs having no identified HF clinics, and a wide range in the population served by each clinic. As anticipated, the clinics were varied in structure and the services offered. The greatest variation in terms of intensity and complexity was in terms of the education service offered. Remote monitoring and a home-base component to the HF clinic services were notably absent in most clinics.

Multi-disciplinary ambulatory complex disease management clinics are increasingly studied as the preferred modality of ambulatory care delivery for chronic diseases such as HF [1,3,4,6,17]. Advocates of such clinics highlight the many randomized clinical trials that show the efficacy of such clinics in reducing mortality and rehospitalisation [3,18-31]. Importantly, although these clinics are grouped together in systematic reviews and meta-analyses, there is heterogeneity in the models evaluated and services offered [7]. Prior to implementing these clinics in routine practice, it is critical to understand which components are central to the intervention. Several meta-analyses have attempted to address this research question using the published literature [3,18-22,27,28]. McAlister and colleagues evaluated 29 trials enrolling a total of 5,039 patients [3]. Because of substantial heterogeneity, they did not report an overall summary statistic [3]. They found that multi-disciplinary clinics improved mortality, while tele-monitoring improved re-hospitalization rates [3]. Holland and colleagues contrasted studies that incorporated home visits, or between visits telephone calls, to those that were solely hospital or clinic based [27]. In the 30 trials that were included in their analysis, they found that reductions in hospitalization were limited to studies that included either a home-based or telephone based component to the intervention.

Our study provides a number of insights for policy makers who are planning the implementation of such disease management systems in other regions. The proliferation of heart failure clinics in Ontario has occurred without specific guidance as to their structure, in part due to the absence of dedicated funding. This has resulted in considerable variation in important components such as education, and the notable absence of key features such as a home-based component or remote monitoring. Our findings are consistent with that seen by Driscoll and colleagues who found substantial variation in the care provided at HF management programs across Australia, raising concerns about the quality of care provided to these patients [32].

Understanding the association between heterogeneity in clinic model and outcomes such as mortality and re-hospitalization is the logical next step in order to address if quality of care is compromised by this variation in care models. In patients discharged after a HF hospitalization who were treated at HF clinics, we observed a 1-year mortality of 22.8% and a 1-year rehospitalisation rate for HF of 44.2%. There was a striking 1.5 fold variation in mortality between clinics and a 2.5 fold variation in re-hospitalization rates. This highlights the need to identify which clinic-level components are predictive of improved outcomes, such that one can provide clinicians and policy-makers clear guidance when designing specialized HF clinics. These are foci of further research for our group.

Disease management through specialized HF clinics is recommended by guidelines for patients recently hospitalized with HF or at high risk for decomposition [6,17]. Currently, there is a paucity of data on what proportion of these patients are indeed seen at HF clinics. Although, this study was not designed to address this question, based on our estimates of annual HF discharges in the province and the annual number of new patients seen in HF clinics, it is likely that an only small proportion of appropriate patients are cared for at HF clinics. This is consistent with data from Australia, which suggests only 20% of eligible HF patients are seen at specialized HF clinics [32]. In addition, the catchment area served by each HF clinic (353,800 persons) in our study is larger than that seen in others surveys, such as one in Denmark (1 HF clinic per 115,000 persons) suggesting that there is less access in Ontario compared to other regions [32,33]. Moreover, our environmental scan suggests that there is substantial variation in access to HF clinics across the province. The absence of specific MOTHLC funding for the HF clinics may be a contributing factor. Elucidation of the underlying mechanisms for this disparity will be important for policy makers.

Our study must be interpreted in the context of several limitations. First, although we used a number of different methods to locate all HF clinics in the province, we cannot confirm that all clinics were in fact identified. We used an instrument to evaluate intensity and complexity; this did not cover all potential service components. Indeed, it does not include post-discharge planning, which has been identified by some studies as a critical component to reduce early rehospitalisation. Finally, although we have categorized clinics into intensity strata based on expert opinion, the relevance of such categories is dependent on their association with improved patient outcomes.

In summary, through our environmental scan, we found that despite the absence of specific governmental funding, there are at least 34 HF multidisciplinary clinics in operation in the province of Ontario. These clinics have a wide range of services offered. Further research on understanding which of these service components are associated with improved patient outcomes will aid policy makers and clinicians to determining the optimal care model for these complex patients.

## Competing interests

None of the authors have any conflicts of interest to declare.

## Authors’ contributions

HCW Conception, design, acquisition, analysis and interpretation of data; drafting of manuscript; final approval of manuscript submitted. GT Design, acquisition and analysis of data; drafting of manuscript; final approval of manuscript submitted. LA Acquisition and analysis of data; revising of manuscript; final approval of manuscript submitted. NM Conception, design, analysis and interpretation of data; drafting of manuscript; final approval of manuscript submitted. WW Acquisition of data; revising of manuscript; final approval of manuscript submitted. MP Analysis, and interpretation of data; revising of manuscript; final approval of manuscript submitted. GvV Conception and design; revising of manuscript; final approval of manuscript submitted. KK Conception and design; revising of manuscript; final approval of manuscript submitted. MK Conception, design, analysis and interpretation of data; revising of manuscript; final approval of manuscript submitted. All authors read and approved the final manuscript.

## Pre-publication history

The pre-publication history for this paper can be accessed here:

http://www.biomedcentral.com/1472-6963/12/236/prepub

## Supplementary Material

Additional file 1 **Appendix A.** Heart Failure Clinic Stratification using Concept Mapping. Click here for file
