# Supplementary material for: Specialized multi-disciplinary heart failure clinics in Ontario, Canada: an environmental scan
Source: BMC Health Serv Res. 2012 Aug 3;12:236. doi: 10.1186/1472-6963-12-236 (PMC3506498; doi:10.1186/1472-6963-12-236)
Supplement: Additional file 1 — Appendix A. Heart Failure Clinic Stratification using Concept Mapping. [file 1472-6963-12-236-S1.doc]

Appendix A:

**Heart Failure Clinic Stratification using Concept Mapping**

Once the surveying of all the clinics was completed, this categorization process was revealed to be complex and nuanced. The initial conceptualization was that the clinics would be categorized by complexity and intensity of intervention. While the HF-DMSI developed by Riegel and colleagues is useful in capturing the richness of the multi-attribute, multi-domain activities of a heart failure clinic, it is not an instrument which provides a summary score that could be used to rank the clinics.(12) To overcome this limitation, a concept mapping exercise was conducted consisting of two parts.

The first part was a priming exercise where the relative importance of the measured elements of the HF-DMSI was determined by a multidisciplinary expert panel. The second part was a categorization of the scored clinics into three intensity levels based on their scores on the HF-DMSI instrument and the implicit weighting of the HF-DMSI elements in the first part of the exercise.

This technique was pioneered by psychologist George Kelly whereby the categorization of many elements could be reliably and stably obtained through and exercise called a "card sort".(13) This technique has been found to be reliable and robust and is used frequently in software design, taxonomy development and other fields where multi-attribute categorization or stratification is required.(14)

There are two types of card sort – open and closed. An open card sort requires that the individual or group create categories themselves from the elements to be sorted – the number of categories is open, as is any labelling of those categories. A closed card sort provided a pre-specified set of categories to which all elements must be assigned.

For the categorization of the heart failure clinics we used a closed card sort in two parts. We gathered a multi-disciplinary panel of experts working in heart disease management and treatment, as well as a several people working in heart failure clinics in different capacities. This provided us with a panel with broad knowledge of the subject area, rich experience and deep, relevant professional knowledge of cardiac care in general and heart failure clinics specifically.

In the first part of the closed card sort we needed to prime the process based on the elements described in the HF-DMSI. For this purpose we decomposed each of the ten elements of the instrument into their descriptive components for individual categorization. For instance, in the element of "Social Support/Peer Support" there are three components; "No mention of a peer support intervention", "Peer support mentioned but not integral to intervention" and "Peer support integral component of intervention". These components, stripped of their scoring values, where then placed by group consensus into the three categories – high, medium and low. Although this process may seem trivial, it often led to splits that were not obvious before the discussion. Especially in the division of four- and five-component elements we found that the development of a consensus was instructive. At the end of the priming exercise we had placed all 38 descriptive components into one of the three categories. This stratification provided the framework for the second part of the card sort.

In the second part of the card sort we utilized the concepts mapped in the first part to inform the categorization of each clinic into High, Medium and Low service categories. To do this we took each clinic, identified only by a random number, and created based on scores on the HF-DMSI, a narrative description of that clinic, using the descriptive components that were actually ascribed to those clinics in the survey process. This meant that the same set of descriptive attributes categorized in the first part of the exercise where used as markers of clinic activity for the purposes of categorization. For simplicity, the capsule descriptions only included eight of the 10 elements from the HF-DMSI, as all 30 clinics shared the same scores on two elements. We then assigned each clinic to one of the High, Medium or Low service strata through a consensus-generating process.
